# Gender-sensitive and diversity-sensitive end-of-life care in Switzerland (GiveCare): a mixed-methods study protocol

**DOI:** 10.1136/bmjopen-2026-120065

**Published:** 2026-06-28

**Authors:** Bettina Schwind, Andri Tschudi, Carmen Sant Fruchtman, Andrea Schöpf-Lazzarino, Katarina Kebis, Elke Steudter, Frank Luck, Daniel Cobos Muñoz, Caroline Hertler, Karin Ribi

**Affiliations:** 1 Careum School of Health, Kalaidos University of Applied Sciences, Zurich, Switzerland; 2 Department of Epidemiology and Public Health, Swiss Tropical and Public Health Institute, Allschwil, Switzerland; 3 University of Basel, Basel, Switzerland; 4 Catholic University of Applied Sciences Freiburg, Freiburg, Germany; 5 Competence Center Palliative Care, Department of Radiation Oncology, University Hospital Zurich, Zurich, Switzerland

**Keywords:** PALLIATIVE CARE, Caregivers, Health Equity, Health Services, Patients, Hospitals

## Abstract

**Abstract:**

**Introduction:**

Palliative care has been identified as one of the most inequitable areas of healthcare. In Switzerland, as globally, disparities in end-of-life care (EOLC) exist along socio-demographic lines, shaping the access to and quality of care received across services by patients and their caregivers. Research has linked these disparities to binary gender differences and other aspects of a person’s social position. The GiveCare project aims to provide a systemic understanding of how aspects of gender and diversity intersect to shape the provision of EOLC in Switzerland and to translate the generated knowledge into practice, policy, education and training.

**Methods and analysis:**

GiveCare employs a sequential mixed-methods design, combining a feminist intersectional approach with a systems thinking lens. It consists of four work packages (WPs): (1) A national survey will assess the perceived awareness of gender and diversity among palliative care professionals. (2) A focused ethnography will provide in-depth insights into the care journeys of patients and their significant others in two specialised inpatient palliative care units. (3) A social network analysis and discrete event modelling will unpack the complexity of such care journeys across inpatient and outpatient settings in the Canton of Zurich. 4. Throughout the process, an integrated knowledge translation approach will help to generate actionable evidence, co-created with patients, caregivers, professionals and policymakers, to enhance inclusivity and equity in EOLC practice and policy.

**Ethics and dissemination:**

The Ethics Committee of the Canton of Zurich, Switzerland, granted ethical approval for WP2–4 (Req-2025-02241) and issued a waiver for WP1 (Req-2025-00211). The research team will conduct the research in accordance with the Swiss Federal Act on Data Protection and the rules and regulations of the Swiss Federal Data Protection and Information Commissioner. The findings will be disseminated through peer-reviewed publications and conference presentations as well as via the community of practice established through the integrated knowledge translation process.

**STRENGTHS AND LIMITATIONS OF THIS STUDY**

- The study combines an intersectional approach with a systems thinking lens to provide comprehensive evidence of how gender and diversity shape end-of-life care.
- Integrated knowledge translation involves potential knowledge users in the research process from the outset, enabling them to contribute to and benefit from the research at every stage.
- The sequential mixed-methods design combines survey data, ethnographic inquiry and systems analysis to capture the complex, multilevel dynamics of end-of-life care without oversimplifying diverse care relations and journeys.
- The in-depth ethnography and systems analysis focus on specialised palliative care and one canton, respectively, which may not fully capture the range of end-of-life-care experiences in Switzerland.
- Recruiting seriously ill patients and their significant others may be challenging due to unpredictable illness trajectories and clinician gatekeeping.

## Introduction

 The global burden of serious health-related suffering requiring palliative care (PC) is projected to increase by 57% in high-income countries between 2016 and 2060.^1^ In Switzerland, a high-income country with a fragmented healthcare system,^2^ this projection raises pertinent questions about the best ways to support people in the final phase of their lives. PC is a fundamental human right that should guarantee dignity and alleviate suffering for all, irrespective of gender, location or socioeconomic status.^3^ However, access to PC is highly inequitable globally, with factors such as age, gender, belief, disability and socioeconomic status having an impact.^4^ Even in countries where access to PC is well established, research shows that significant inequities persist due to systemic and structural disparities.^5 6^

These overall inequities are also evident in research showing binary gender disparities in end-of-life care (EOLC),^7^ a key component of PC aimed at individuals likely to die within 6–12 months.^8^ Women report more symptoms and less improvement in quality of life after initiating PC compared with men. They are also more likely to seek help and activate extended support networks, while men tend to experience depression and increased dependence on their significant others.^7^ Such disparities reflect societal norms that influence the expectations and behaviours of professionals, patients and caregivers alike,^9 10^ meaning that gendered ideas such as heteronormativity persist in health and social care.^11^

These gendered norms extend beyond formal healthcare settings. The majority of PC is provided by family caregivers (75%–90%),^12^ and Canadian data suggest that 77% of the PC costs are attributable to unpaid care work.^13^ A disproportionate amount of this unpaid care is provided by women.^14^ Female caregivers are more likely to take on caregiving roles, juggle multiple responsibilities and receive less external support.^15^ Men also provide care for dying partners, but this can create confusion about how to reconcile caregiving with socially expected ways of being a man.^16^ At the same time, older people are reported to exhibit flexibility in gender roles,^17^ suggesting that societal assumptions about gender and caregiving may not fully capture the diversity of caregiving experiences.^11^ Despite the central role of family members in EOLC,^18^ their needs are often insufficiently addressed in clinical practice.^19^ Support for families is often limited to specific settings, which can result in gaps in caregiver involvement during transitions or discharge.^20^

These gaps in recognising and supporting caregivers are further compounded for groups who already experience marginalisation in healthcare. Gender and sexually diverse people (LGBTQIA+) may face additional challenges related to discrimination and hetero-/cisnormative assumptions by providers,^10^ and have reported receiving inadequate, disrespectful or even abusive care.^21^ Individuals with multiple intersecting marginalised social positions may experience even greater discrimination and disadvantage, resulting in diverse EOLC needs and preferences.^11^

Against this backdrop, the implicit biases of health and social care professionals can influence the provision of care in ways that are detrimental to certain groups, potentially resulting in gender or other diversity-related disparities.^22 23^ Providing appropriate EOLC to patients from diverse backgrounds thus requires awareness of social, economic and cultural variability, as well as critical reflection on healthcare professionals’ own stereotypes and biases regarding ethnicity, gender, religion and spirituality.^24^ Although PC core competencies require such self-awareness and reflection,^25^ a scoping review revealed that PC professionals lack knowledge and understanding of diverse backgrounds, and insufficiently reflect on prejudices, biases and anxieties about culturally different beliefs and practices.^26^

Although the importance of gender-sensitive and diversity-sensitive holistic EOLC is growing,^27^,^31^ gender continues to receive little attention in PC research, practice and policy.^32^ Inequalities are mainly reported in relation to binary understandings of gender or other single factors that shape a person’s social position.^11^ Consequently, there are discrepancies between the aims of holistic EOLC and the lived experiences of people who do not fit the current PC system, particularly those situated at intersecting marginalised positions.^11 33^ Similarly, little is known about how aspects of gender and diversity affect the role of caregivers in EOLC, or how they receive interprofessional support in inpatient and outpatient settings. To develop innovative strategies to reduce disparities, we need a better understanding of the factors that contribute to inequality in access and provision of EOLC in the Swiss context. This requires an intersectional approach to explore the influence of gender and other social factors on individual and group disparities.^11 34^

Against this background, the overall objectives of this research project are (1) to comprehensively understand how aspects of gender and diversity are shaping the provision of EOLC, and (2) to translate the generated knowledge into practice, policy, education and training. Specifically, the study explores how EOLC professionals perceive their awareness of gender and diversity, and what kind of gender-specific and diversity-specific gaps and opportunities exist in current EOLC across inpatient and outpatient settings.

## Methods and analysis

### Theoretical framework

The research project draws on the following theories and concepts as complementary lenses for understanding gender and diversity in EOLC: First, we use a multilevel approach to examine gender and diversity across individual (patient and caregiver), interpersonal (healthcare team), organisational and policy levels.^35^ Second, we employ a systems thinking lens to analyse formal and informal networks of care and the continuum of services^36^ in order to capture the range of interactions and unintended consequences in EOLC. Third, a feminist intersectional approach allows us to move beyond binary notions of femininity and masculinity to explore the interactions between multiple, intersecting dimensions of gender and diversity.^37^,^39^ We combine this approach with Bourdieu’s^40^ concepts of ‘field’, ‘capital’ and ‘habitus’ to elucidate how social categories and hierarchies are created and reinforced within EOLC settings.

### Study design

**Figure 1:**
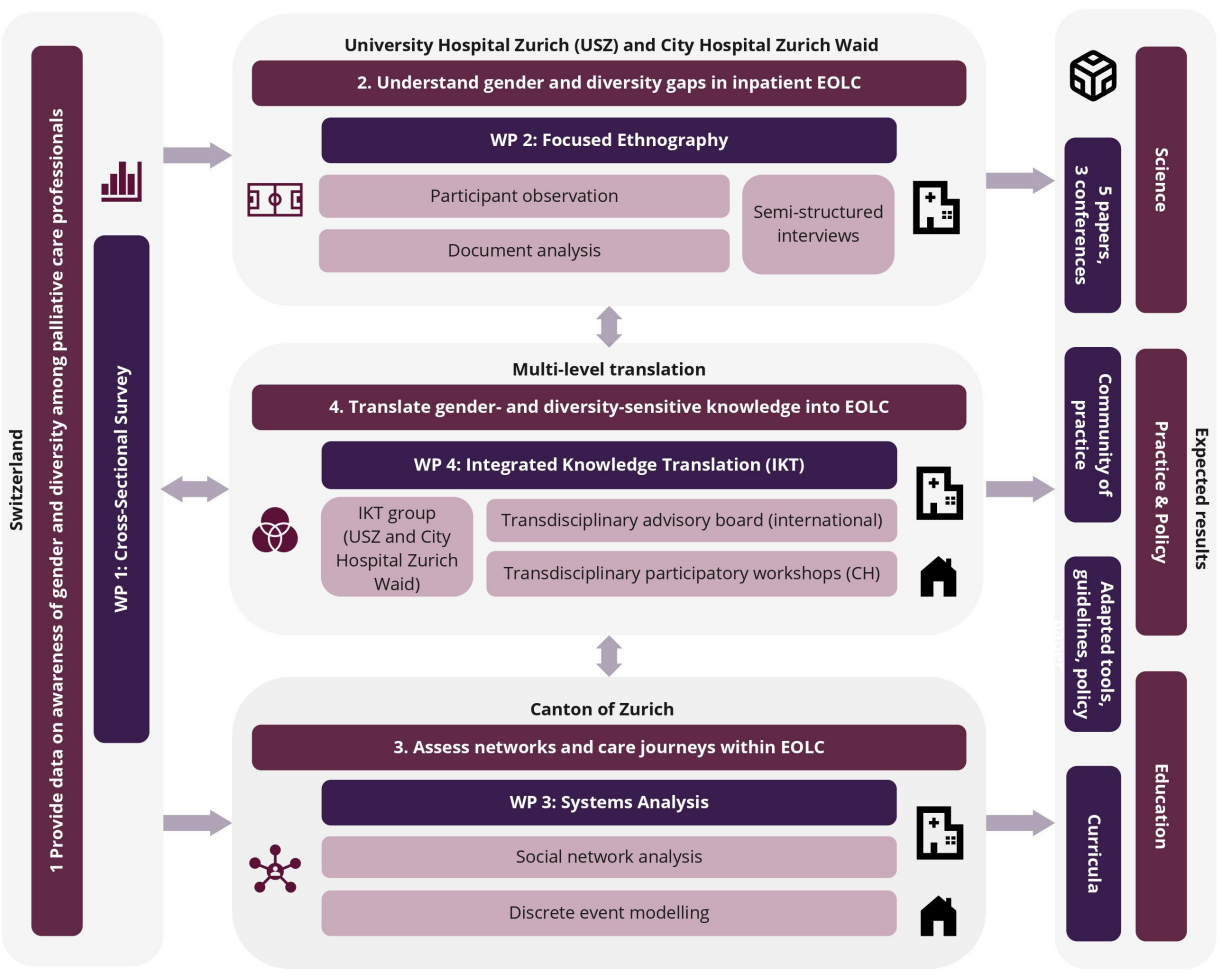
Overview of the GiveCare study design. The study integrates four interlinked work packages (WPs): WP1 surveys palliative care (PC) professionals’ gender-awareness and diversity-awareness; WP2 explores inpatient end-of-life care (EOLC) practices ethnographically; WP3 maps care networks and journeys; and WP4 translates co-created findings into practice, education and policy through an integrated knowledge translation (IKT) process.

The research project started in April 2025 and will be completed by March 2028. It applies a sequential mixed-methods design^41^ consisting of four interlocking work packages (WPs): In WP1, a Swiss-wide cross-sectional online survey will provide data on PC professionals’ gender and diversity awareness. In WP2, a focused ethnography will explore the gender and diversity gaps and opportunities in inpatient EOLC on the individual, interpersonal and organisational level. In WP3, a system analysis will assess networks and care journeys across inpatient and outpatient EOLC on the interpersonal, organisational and systems level. The WPs are framed by an integrated knowledge translation process (IKT), WP4. The IKT will ensure that knowledge users are involved in the research process from the outset enabling emerging insights to be integrated into EOLC in real time. The WPs are detailed in figure 1.

### Study settings

In WP1, we will assess PC professionals’ gender and diversity awareness on a national level, capturing similarities and differences across language regions and professional groups. In WP2 to WP4, we focus on the Canton of Zurich and its surrounding areas. Concentrating on one region allows for a comprehensive understanding of the subject matter and ensures the feasibility of the study. WP2 and WP3 will concordantly be conducted in partnership with the Competence Centre for Palliative Care (CCPC) at the University Hospital Zurich (USZ) and the City Hospital Zurich Waid. The close collaboration with relevant stakeholders from these clinical institutions will facilitate the effective implementation of the IKT process. The CCPC at the USZ is specialised in the comprehensive treatment and care of adults with advanced, incurable diseases. It provides inpatient specialised PC for approximately 377 patients per year,^42^ offers about 507 interprofessional consultations for hospitalised patients per year^43^ and delivers around 160 interprofessional consultations for outpatients per year.^44^ Most patients are diagnosed with oncological conditions (80%), while a minority suffers from other severe illnesses (20%).^42^ According to our clinical partners, the palliative care unit at the City Hospital Zurich Waid records around 300 hospitalisations per year. Because the hospital is one of the most common places of death in Switzerland,^45^ these settings are well suited to explore transitions and links between inpatient and outpatient EOLC.

### WP1: National cross-sectional online survey

The aim of WP1 is to assess PC professionals’ perceptions of gender-sensitive and diversity-sensitive care through a national cross-sectional online survey.

#### Sampling and recruitment

The survey ran from the end of November 2025 to March 2026. Survey participants were professionals who provided PC in Switzerland from various professions (eg, medicine, nursing, social work, psychology and spiritual care) and settings (eg, inpatient acute care setting, outpatient setting).^46^ Recruitment relied on the support of stakeholders who have endorsed the GiveCare project and contacted other stakeholders (eg, family doctors) to distribute the online survey link through their communication channels. In addition, we contacted institutions that provide PC, as well as professional associations and networks. We also recruited via the social media platforms LinkedIn, Facebook and Instagram.

#### Data collection

The survey comprises four parts. First, we collected data on sociodemographic (eg, gender, age, nationality, profession, education) and workplace characteristics (eg, region, setting). Second, we assessed the PC professionals’ awareness of key social factors shaping EOLC: gender, socioeconomic status, education and age. To this end, we adapted questions from the Nijmegen Gender Awareness in Medicine Scale (N-GAMS)^47^ to cover awareness and self-assessed knowledge of all four factors. Third, we evaluated the extent and strength of interprofessional collaboration across EOLC settings. Fourth, we included a social network assessment to collect data for the social network analysis (SNA; see section ‘WP3: System Analysis’). The survey items were translated into German, French and Italian. The survey was carried out online using the open-source mobile data collection platform ODK.^48^

#### Data analysis

Data will be analysed with SPSS v25 (SPSS Inc., IBM, IL USA). Guided by a descriptive intercategorical approach,^49^ we will examine how gender-sensitivity and diversity-sensitivity scores vary across groups defined by intersectional positions. Descriptive statistics will include classifications in contingency table and tests of difference (T-tests, Kruskal-Wallis or analysis of variance (ANOVA)) depending on the numbers for each classification.^49^ Regression analysis that allows for heterogeneity across intersections (eg, regression with interactions) may also apply. The exact analysis proceedings will be defined based on the obtained data sample and discussed with the advisory board, the IKT group (see section ‘WP4: Integrated Knowledge Translation (IKT)’) and a statistician familiar with this type of analysis.

### WP2: Focused ethnography

The aim of WP2 is to explore how gender and diversity shape EOLC by examining the perspectives of providers, patients and family caregivers, and by observing how care is practised and documented throughout the inpatient EOLC journey, including transitions to other settings. We use focused ethnography (FE) because it offers a powerful tool for qualitatively exploring power dynamics and social relations in healthcare settings.^50^ FE avoids additive categorical thinking,^51^ engages key (insider) informants to generate practice-relevant insights,^50 52^ and supports informal exchange and reflexive attention to intersectionality. This methodological flexibility makes FE particularly well suited to the sensitive EOLC setting.^51^

#### Sampling and recruitment

Local research coordinators will screen all patients treated at the CCPC and the City Hospital Zurich Waid to identify potential participants. Patients should have a life expectancy of up to 1 year, assessed by the surprise question used to identify patients nearing the last phase of life.^53^ If this criterion applies and is confirmed by the involved member of the clinical team, we will apply criteria-based purposive sampling to address intersectional issues defined with the advisory board and the IKT group. For each participating patient, we will ask a significant other (caregiver, family member, friend) to participate. We plan to include 15–20 patient-significant other dyads, as code and meaning saturation may be achieved with 16–24 interviews.^54^ Interviewed professionals need to be directly involved in the care of the recruited patients. Selection will also follow criteria-based purposive sampling to ensure that all types of professions providing inpatient EOLC are included.

#### Data collection

We will collect data over a period of approx. 6 to 9 months using three complementary methods: (1) participant observation, (2) semistructured interviews and (3) document analysis.

Observations will take place during three key clinical situations that reflect the inpatient EOLC journey, including the transition to other settings, home or death: (1) the clinical entry assessment; (2) the round table for complex situations; and (3) the exit interview. Data collection will be based on a semistructured observation tool, guided by the SENS^55^ and the checklist for migration sensitive PC.^56^ The SENS—an acronym for symptom management, end-of-life decisions and expectations, network-organisation and support of the carers—is a practice-oriented grid for the assessment, treatment planning and evaluation of life-limiting illnesses, which provides a thematic structure highlighting points at which gender and diversity play a critical role. The observation will include the description of the meeting, participants, interactions, challenges and topics covered. Observers will reflect and note on how inequalities manifest and on similarities and differences between the social positions of those involved.^51^

Semistructured interviews are planned to take place separately with each patient, their significant other and one professional involved in care during the inpatient stay or up to 3 weeks thereafter. Interviews will explore the perspectives and experiences of patients, significant others and professionals regarding the ongoing EOLC journey and (possible) transitions. Interview results will also inform the system analysis in WP3. Interview guides will be pretested and refined as needed during data collection.^51^ Researchers will reflect on how interview dynamics and their own positionalities may implicitly shape experiences of marginalisation and privilege.^51^

Document analysis will assess to what extent gender-related and diversity-related issues are documented in medical charts, and if available, other forms of documentation (eg, meeting protocols) based on a set of predefined criteria^57^ developed with the IKT group (see section ‘WP4: Integrated Knowledge Translation’).

#### Data analysis

We will transcribe and pseudonymise all interviews and analyse observational notes, interview transcripts and documents using reflexive thematic analysis.^58^ The IKT group will regularly review the data and identify themes with analytic rigour achieved through investigator triangulation.^59^ Triangulation aligns well with intersectionality and ensures rigour across levels of analysis by combining multiple methods and data sources.^51^

### WP3: System analysis

The aim of WP3 is to map and analyse the care journeys and relationships of patients and their significant others within EOLC networks across inpatient and outpatient settings in the Canton of Zurich, using SNA and discrete event modelling.

#### Sampling and recruitment

For WP3, we will recruit participants through two recruitment pathways, each associated with a specific data collection method. First, we will invite patients from WP2 who agreed to be re-contacted, as well as additional patients screened by the local research coordinators. Participants will be asked to identify the members of their care network in a short, structured survey. Using snowball sampling, we will reach out to the identified members and invite them to complete the survey as well.

Second, in collaboration with the local research coordinators, we will recruit additional patients, significant others and professionals for focus group discussions (FGDs). Purposive sampling—guided by findings from WP1, WP2 and preliminary SNA results—will ensure diversity and relevance of perspectives.

#### Data collection

Data collection will include: (1) SNA survey questions (from WP1), (2) a short, structured survey and (3) FGDs.

First, we integrated SNA questions into the EOLC professional survey (see section ‘WP1: Cross-sectional online survey’). Items explore professional roles and frequency of contacts across departments, institutions and sectors to assess network structure, strength and gaps.

Second, identified patients and members of their care network will complete a short (<15 min) survey assessing the nature, frequency (daily, monthly, yearly) and strength of ties within their support network. Based on an intercategorical approach,^60^ the survey will also capture sociodemographic information on age, gender, nationality, socioeconomic status, education and living situation. Participants may complete the survey by phone, via Zoom/Teams or through an individual online link, using the secure ODK platform.^48^ A small group of patients and significant others (2–4 persons) who are not part of the study will pilot-test the survey to ensure clarity and feasibility.

Third, we plan to conduct approximately 7–8 FGDs to interpret SNA findings and to explore how social and gender norms shape EOLC network structures. Members of the research team will facilitate two FGDs with patients (3–6 participants each), two FGDs with significant others (5–6 participants each) and three to four FGDs with formal care providers (5–6 participants each). Using a participatory network mapping approach,^61^ participants will reflect on the visualised social networks obtained from survey data with a focus on social and gender norms. FGDs will last 60–90 min and will be video recorded.

#### Data analysis

From an intersectional perspective, we will use standard quantitative methods^62^ to identify how gender and other sociodemographic dimensions influence network dynamics.^62 63^ The results will be converted into two adjacency matrices: one representing the care networks of patients and one representing the professional networks. We will import the tables into Gephi^64^ to produce the network visualisation and to calculate key metrics of the EOLC network, including size, density and cluster coefficients, as well as node-level measures such as centrality, betweenness and closeness.^65^ These metrics will allow us to identify gender-based and diversity-based differences in communication patterns, influence and support within care networks.

Building on these findings as well as those from WP1 and WP2, we will develop a discrete event model using discrete event simulation to understand the end-to-end patient pathway through the EOLC system.^66^ Based on the qualitative data of WP2, we will apply thematic analysis to identify and visualise main barriers, inefficiencies and bottlenecks across inpatient and outpatient care pathways.^67^ We will develop several discrete event models to reflect common care trajectories among different patient groups. In addition, experienced systems researchers will create process maps visualising the patient pathway using Standard Business Process Mapping Notation (BPMN) 2.0.^68 69^ These maps will include: entities (eg, patients and significant others), attributes (eg, age, gender), events (eg, visit PC unit), decision points (eg, referral) and resources involved in delivering care.^69^ The process map will depict the sequence of activities enacted by PC professionals (medicine, nursing, social work, psychology and spiritual care) in their interactions with patients and their significant others in EOLC.^70^ Data from the SNA surveys, patient network surveys and focus group discussions will be aggregated in a discrete event model to understand patients’ and significant others’ journeys through the system from an intersectional perspective. Findings from WP3 will be discussed in two transdisciplinary participatory workshops with system stakeholders.

### WP4: Integrated knowledge translation (IKT)

We will use IKT to translate project findings into practice in real time. IKT is a participatory, non-linear approach that promotes dynamic partnerships between researchers and knowledge users (eg, clinicians, nurses).^71^ It aims to transcend and integrate knowledge across disciplinary and sectoral boundaries.^72^ By spanning the entire research process, IKT enables the immediate co-creation and application of findings to practice and policy. Our approach is guided by the recommendations from the Canadian Institutes of Health Research^73^ and is structured around three core elements: an advisory board, an IKT group and participatory workshops.

#### Advisory board

A transdisciplinary advisory board of experts and stakeholders will guide the GiveCare project. It will provide input and advice at pre-determined intervals and support the development of a community of practice.^74^ We aim to establish a diverse consortium of members from established research networks and specialised interprofessional organisations in EOLC. These members will provide interdisciplinary and transdisciplinary advice, support implementation efforts and champion knowledge dissemination—for example through conference contributions, working groups and integrating findings into professional guidelines or educational curricula. Through this collaborative structure, we expect to organically build a wider community of practice in Switzerland and beyond, advancing gender-sensitive and diversity-sensitive EOLC.

#### IKT group

We will establish an IKT group to support real-time knowledge generation and implementation in collaboration with our clinical partners, the CCPC at the USZ and the City Hospital Zurich Waid.^71 75^ The group will include researchers (principal investigator, co-investigators, ethnographers), point-of-care clinicians (physicians, nurses, social workers, spiritual counsellors, psychologists etc), as well as patients and significant others. Involving patients and significant others is essential when evaluating new PC approaches, as healthcare providers’ perspectives often take centre stage.^76^ At a kick-off meeting, we will introduce GiveCare to potential knowledge users and explain IKT principles, enabling them to decide whether and in what capacity they wish to participate. Roles and responsibilities will be defined in a partnership agreement.^73^ Regular meetings will facilitate shared decision-making regarding participant recruitment, data collection (eg, choice of observation settings) and the translation of findings into clinical practice.^71^ Together with the IKT group, we will establish reflexive communication and feedback processes aimed at developing concrete solutions to promote gender-sensitive and diversity-sensitive inpatient EOLC (eg, adaptation of the SENS or other tools and integration of reflexive processes).

#### Participatory workshops

We will conduct two transdisciplinary participatory workshops to discuss the SNA visualisations and to review and critique the discrete event model developed in WP3 until consensus is reached. The workshops will allow to identify practical solutions for the promotion of gender-sensitive and diversity-sensitive EOLC from a health systems perspective.^77^ They will provide a platform for knowledge exchange and co-creation, bringing together policymakers and diverse EOLC stakeholders to foster dialogue, identify opportunities for implementation and define next steps. These workshops will help ensure that gender and diversity considerations are effectively disseminated and integrated into the strengthening of the EOLC system, using the Canton of Zurich as example.

## Patient and public engagement

The study design evolved through discussions with our clinical partners, the CCPC at the USZ and City Hospital Zurich Waid. The IKT process further integrates EOLC professionals, patients and their significant others as knowledge users into the research process. The participatory workshops will provide the opportunity to expand the range of stakeholders to include policymakers. During data collection, knowledge users will review data collection tools, suggest relevant directions of inquiry, support recruitment and help define sampling criteria. Knowledge users will also repeatedly review data, identify emerging themes in the data analysis and disseminate findings (see section ‘Ethics and Dissemination’).

## Ethics and dissemination

The Ethics Committee of the Canton of Zurich, Switzerland, reviewed the research project and granted ethical approval for WP2–4 (Req-2025-02241). The committee did not consider WP1 to fall under the Swiss Law on Human Subjects research and issued a waiver (Req-2025-00211). Ethical procedures vary according to the data collection methods used. All participants in WP2 and WP3 will provide written informed consent before data collection begins. Participants in WP4 will not provide written informed consent, since the data collected concerns group perspectives rather than person-specific data. Instead, we will ask for their verbal consent to keep the content of the discussions confidential.

We will record interviews and FGDs and transcribe the recordings verbatim using a transcription service or a Swiss cloud software. The transcripts will be pseudonymised and securely stored on local network folders. The recordings will be deleted after transcription. Due to the group setting, participants will be informed that absolute confidentiality cannot be guaranteed during FGDs, and they will be reminded to respect the privacy of other group members. We will record data from observations and document analysis on predefined electronic recording sheets without including any sensitive identifying information. SNA survey data will be collected using the secure Open Data Kit (ODK) platform.^48^ Names and contact details will be recorded on a separate participant identification list. No personal (or identifiable) data relating to participants will be published.

We plan to disseminate research results by submitting 4–6 publications to peer-reviewed journals, presenting them at three conferences and publishing two blog posts. Through the IKT process, we aim to establish a community of practice for gender-sensitive and diversity-sensitive EOLC. Professionals in research, practice and education will disseminate co-created findings in formats relevant to their field of practice (eg, policy paper, guidelines, curricula) using their networks and communication channels.

## Discussion

The research project is expected to generate comprehensive, multilevel evidence of how gender and diversity shape EOLC in Switzerland. Using a sequential mixed-methods design, GiveCare will provide first-hand data on the current state of professionals’ awareness of gender-sensitive and diversity-sensitive EOLC. The focused ethnography will complement this data by providing in-depth qualitative insights into current inpatient EOLC practice. From a systems thinking perspective, SNA and discrete event modelling will enable a detailed examination of EOLC processes and care networks across inpatient and outpatient settings.

A major strength of the research project lies in its multilevel design and use of a systems thinking approach. Traditionally, frameworks in EOLC have focused on patient-level factors.^78^ By connecting individual, interpersonal, organisational and policy levels,^35^ GiveCare goes beyond a narrow patient-centred focus to include the perspective of significant others and to explore how patients and significant others are embedded in broader systems of care, thereby fully capturing the range of interactions and consequences emerging from within such systems.^36 79 80^ This makes GiveCare well suited to addressing the complex interplay of individual, social and systemic factors that characterise EOLC.^79 81^

The use of intersectionality is another strength of the research project. Intersectional approaches still remain uncommon in health research,^34 82^ and we therefore lack an understanding of how gender interacts with other factors in creating disparities in EOLC.^83^ By looking at gender in its intersection with various axes of inequality, GiveCare has the potential to identify and address marginalising practices in EOLC, thereby promoting equity and fostering innovation.^84^ Considering gender in its interaction with other factors of inequality also closely reflects real-life complexities,^34^ yielding insights that are directly relevant to clinical practice.

The IKT process further strengthens the link between research and clinical practice. Involving knowledge users in the research process facilitates findings that are relevant to practice and policy and creates a platform for implementation strategies.^85^ Importantly, the benefits of the approach are not limited to actionable outcomes. The process itself is transformative by creating a new community of practice and collaborations across fields and professional roles that may have an effect beyond the research project.^86^

The research project also faces several challenges. Operationally, the sequential mixed-methods design, which involves several interlocking WPs, is complex and involves researchers from different institutions and with various disciplinary backgrounds, potentially leading to heterogeneity in data collection and analysis. To mitigate heterogeneity and provide cohesion across the WPs, we will adhere to the theoretical framework throughout the research process. Continuous exchanges involving bi-weekly team meetings and bi-yearly extended reflexive workshops will further facilitate efficient coordination and increase reflexive awareness of how researchers’ own positionalities and perspectives influence data gathering and interpretation.

In addition, the end-of-life situation is inherently sensitive, presenting ethical and recruitment challenges.^87 88^ Doing research with patients at the end-of-life requires being highly attentive to power imbalances and paying close attention to open and sensitive questioning, the timing and circumstances of interviewing and the involvement of significant others.^89^ To this end, we will ensure careful briefing and de-briefing of researchers and closely collaborate with patients, significant others and point-of-care professionals to identify ethical concerns early on and to jointly reflect on them and address them where needed.

Recruitment is also known to be challenging in PC contexts due to high attrition rates and clinician gatekeeping as clinicians may seek to protect patients seen as vulnerable.^90^ With our research design, we mitigate these challenges by recruiting patients in specialised PC centres allowing for a close collaboration with the local research coordinators and clinicians and by using short and flexible data collection periods. However, focussing on specialised PC with a high prevalence of oncological conditions may not fully capture the experiences of patients suffering from non-malignant conditions or whose PC needs remain unrecognised or unmet.^91^

Using an intersectional approach demands a strong reflexive element.^51^ Critically interrogating gender and diversity in EOLC also requires the reflexive examination of how the different positionalities of all people involved in GiveCare—researchers and participants—influence the research process^38 51 92 93^. This reflexive practice allows for the recognition of possible ‘stereotyping’, ‘categorisation’ and ‘culturalisation’ of people (personal and intrapersonal reflexivity). Ethnographic researchers will keep a reflective journal during the interpretation of qualitative data, including a narrative audio-biography and reader-response exercises. We will also engage in collaborative reflexivity by holding structured team-reflexive discussions and facilitating member reflection through the IKT process.^94^
