# Supplementary material for: Gender-sensitive and diversity-sensitive end-of-life care in Switzerland (GiveCare): a mixed-methods study protocol
Source: BMJ Open. 2026 Jun 28;16(6):e120065. doi: 10.1136/bmjopen-2026-120065 (PMC13311621; doi:10.1136/bmjopen-2026-120065)
Supplement: Supplementary file 1 [file bmjopen-2026-120065.reviewer_comments.pdf]

PEER REVIEW HISTORY

BMJ Open publishes all reviews undertaken for accepted manuscripts. Reviewers are asked to complete a checklist review form and are provided with free text boxes to elaborate on their assessment. These free text comments are reproduced below.

ARTICLE DETAILS

Title (Provisional)

Gender-and diversity-sensitive end-of-life care in Switzerland (GiveCare): A mixed-methods study protocol

Authors

Schwind, Bettina; Tschudi, Andri; Sant Fruchtman, Carmen; Schöpf-Lazzarino, Andrea; Kebis, Katarina; Steudter, Elke; Luck, Frank; Cobos Muñoz, Daniel; Hertler, Caroline; Ribi, Karin

---

This article was not externally reviewed at BMJ Open. Protocols that have been independently assessed prior to submission to BMJ Open are usually fast-tracked to publication on the grounds that further substantial changes will not be possible. This independent assessment will usually be external, independent review for both a substantial grant award from a non-commercial or government funder and ethics approval.
